# Supplementary material for: Mathematical Modeling and Robustness Analysis to Unravel COVID-19 Transmission Dynamics: The Italy Case
Source: Biology (Basel). 2020 Nov 11;9(11):394. doi: 10.3390/biology9110394 (PMC7697740; doi:10.3390/biology9110394)
Supplement: Supplementary file 1 [file biology-09-00394-s001.zip › supplementary_file_review/SupplementaryMaterialsMDPI_rev.pdf]

Mathematical modeling and robustness analysis to unravel COVID-19 transmission dynamics: the Italy case

C. Antonini [1], S. Calandrini [1,2], F. Stracci [3], C. Dario [4], F. Bianconi [5]

[1] *ICT4Life Srl, Perugia, Italy*
[2] *Department of Engineering, University of Perugia, Perugia, Italy*
[3] *Department of Experimental Medicine, University of Perugia, Perugia, Italy*
[4] *Regional Government of Umbria, Perugia, Italy*
[5] *COVID-19 epidemiological unit, Regional Government of Umbria, Perugia, Italy*

S1 Conditional Robust Calibration (CRC)

At the end of model calibration, the output of the procedure is the approximate posterior distribution of the parameter vector $f_{\mathbf{P}|P_{O,\epsilon}}$, where $P_{O,\epsilon}$ is the set of parameter samples accepted in the final CRC iteration. Table $S1$ and Table $S2$ show the output of CRC calibration in the last iteration for the Umbria and Italy case, respectively. Both tables report the mode vector of the probability density function (pdf) $f_{\mathbf{P}|P_{O,\epsilon}}$, which is the vector that reproduces the observed data with the highest probability. We also show the 60th, 70th and 90th percentile of the pdf. These values are used to simulate the model time behavior in Figure 2 and Figure 3 of the main file. Moreover, the 90th percentile is used as the range of variation of parameters when running the Conditional Robustness Analysis (CRA).

Table S1: CRC results for Umbria. In the second column, the final mode vector returned by CRC in one of the 10 realizations is shown while the other columns show the 60th, 70th and 90th percentile of the corresponding probability density function (pdf), respectively.

Parameter	p_{mode}	60th percentile	70th percentile	90th percentile
b_e	1.2633	[1.3378-1.3764]	[1.3201-1.3946]	[1.2797-1.4326]
b_0	0.0115	[0.0149-0.0221]	[0.0123-0.0271]	[0.0084-0.0412]
b_1	0.0052	[0.0048-0.0063]	[0.0043-0.007]	[0.0034-0.0088]
b_2	0.0045	[0.0101-0.0162]	[0.0082-0.0203]	[0.0049-0.0323]
b_3	0.0169	[0.0204-0.0326]	[0.0159-0.0425]	[0.0099-0.0699]
FracSevere	0.1131	[0.0841-0.0963]	[0.0790-0.1037]	[0.0678-0.1185]
FracCritical	0.1308	[0.1271-0.1332]	[0.1242-0.1362]	[0.1173-0.1414]
FracAsym	0.2916	[0.2842-0.3030]	[0.2754-0.3137]	[0.2585-0.3314]
IncubPeriod	5.4046	[5.3269-5.4382]	[5.2773-5.5011]	[5.16-5.6316]
DurMildInf	9.9375	[9.8745-10.5087]	[9.5447-10.8324]	[8.8757-11.4873]
DurAsym	10.4581	[10.9705-12.2890]	[10.1745-12.9868]	[8.6730-14.3388]
DurHosp	12.0868	[12.1105-12.6993]	[11.7953-13.0556]	[11.1291-13.9963]
TimeICUDeath	5.7772	[5.4719-5.7225]	[5.3615-5.8587]	[5.1282-6.1768]
ProbDeath	27.0937	[27.2234-28.7913]	[26.4556-29.5931]	[24.4309-31.6999]
PresymPeriod	0.7248	[0.6999-0.7122]	[0.6921-0.7199]	[0.6788-0.7331]
s_{01}	0.5732	[0.5014-0.5286]	[0.4894-0.5446]	[0.4642-0.5730]
s_{02}	0.0955	[0.0735-0.0814]	[0.0697-0.0858]	[0.0631-0.0973]
s_1	0.2360	[0.1788-0.2130]	[0.1659-0.2331]	[0.1420-0.2763]
s_2	0.2481	[0.1666-0.2131]	[0.1507-0.2411]	[0.1230-0.3022]

S2 Conditional Robustness Analysis (CRA)

We run the CRA to understand the impact of epidemiological parameters of COVID-19 and of the implemented intervention measures on the hospitalization rates of Italy and Umbria. For each model parameter, the CRA returns

Table S2: CRC results for Italy. In the second column, the final mode vector returned by CRC in one of the 10 realizations is shown while the other columns show the 60th, 70th and 90th percentile of the corresponding pdf, respectively.

Parameter	p_{mode}	60th percentile	70th percentile	90th percentile
b_e	0.98	[1.0243-1.0550]	[1.0054-1.0729]	[0.9707-1.1067]
b_0	0.0292	[0.0172-0.0233]	[0.0148-0.0268]	[0.0114-0.0353]
b_1	0.0585	[0.0236-0.0361]	[0.0194-0.0428]	[0.0125-0.0655]
b_2	0.0249	[0.0203-0.0297]	[0.0169-0.0355]	[0.0116-0.0505]
b_3	0.0066	[0.0082-0.0156]	[0.0057-0.0201]	[0.0030-0.0413]
FracSevere	0.1066	[0.1061-0.1235]	[0.0988-0.1363]	[0.0814-0.1624]
FracCritical	0.0944	[0.0981-0.1058]	[0.0942-0.1095]	[0.0847-0.1161]
FracAsym	0.3127	[0.3228-0.3539]	[0.3057-0.3698]	[0.2750-0.4068]
IncubPeriod	5.7009	[5.6371-5.9088]	[5.5151-6.0437]	[5.1942-6.3467]
DurMildInf	9.2534	[9.1137-9.9620]	[8.7322-10.4914]	[7.4786-11.4094]
DurAsym	19.8963	[18.26-19.6080]	[17.6938-20.2128]	[16.5383-21.4623]
DurHosp	16.2593	[15.4917-16.7829]	[14.7575-17.4494]	[13.2248-19.0177]
TimeICUDeath	4.8568	[4.7907-4.9548]	[4.7080-5.0873]	[4.5490-5.4549]
ProbDeath	88.6568	[83.4651-85.9289]	[81.9178-87.1277]	[77.7653-89.1394]
PresymPeriod	0.7243	[0.6902-0.7058]	[0.6805-0.7157]	[0.6646-0.7340]
s_{01}	0.5922	[0.5920-0.6158]	[0.5830-0.6293]	[0.5614-0.6568]
s_{02}	0.5035	[0.4935-0.5313]	[0.4785-0.5493]	[0.4449-0.5941]
s_{03}	0.3982	[0.3892-0.4135]	[0.3739-0.4295]	[0.3504-0.4624]
s_{04}	0.1031	[0.0843-0.0987]	[0.0784-0.1052]	[0.0658-0.1208]
s_1	0.1452	[0.1410-0.1587]	[0.1338-0.1680]	[0.1173-0.1892]
s_2	0.1842	[0.1581-0.1898]	[0.1449-0.2097]	[0.1201-0.2477]

in output an index called Moment Independent Robustness Indicator (MIRI), which evaluates the shift between the conditional densities $f_{p_w|L}$ and $f_{p_w|U}$, where $p_w, \quad w = 1, ..., q$ is a model parameter. L and U are, respectively, the lower and upper tail of the evaluation function pdf $f_{Z_i}(z_i)$. Higher is the MIRI value, larger is the shift between the two conditional pdfs. As a result, the perturbation of the parameter space along the direction of the parameter with an high MIRI leads to a substantial variation of the evaluation function. Figures $S1$, $S2$, $S3$, $S4$ show the parameter pdfs $f_{p_w|L}$ and $f_{p_w|U}$ in the two different temporal scenarios of the CRA (until May and until September), for H and ICU variables in Umbria. Figures $S5$, $S6$, $S7$, $S8$ report the parameter pdfs for the Italy case. In blue we represent $f_{p_w|L}$ and in red $f_{p_w|U}$. All figures confirm and better explain the MIRI boxplot depicted in the main text. For example, in Figure $S1$, parameter b_e, $PresymPeriod$, s_{01} and R_0 have a strong separation between the two distributions, which corresponds to a MIRI above 1 in Figure 7 in the main text. By selecting the maximum values of the associated conditional densities, i.e. the mode, we can simulate two totally different behaviors for variable H. On the other hand, parameters with a MIRI around 0, such as b_2, b_3 and $DurAsym$ have overlapping pdfs since their values do not have a

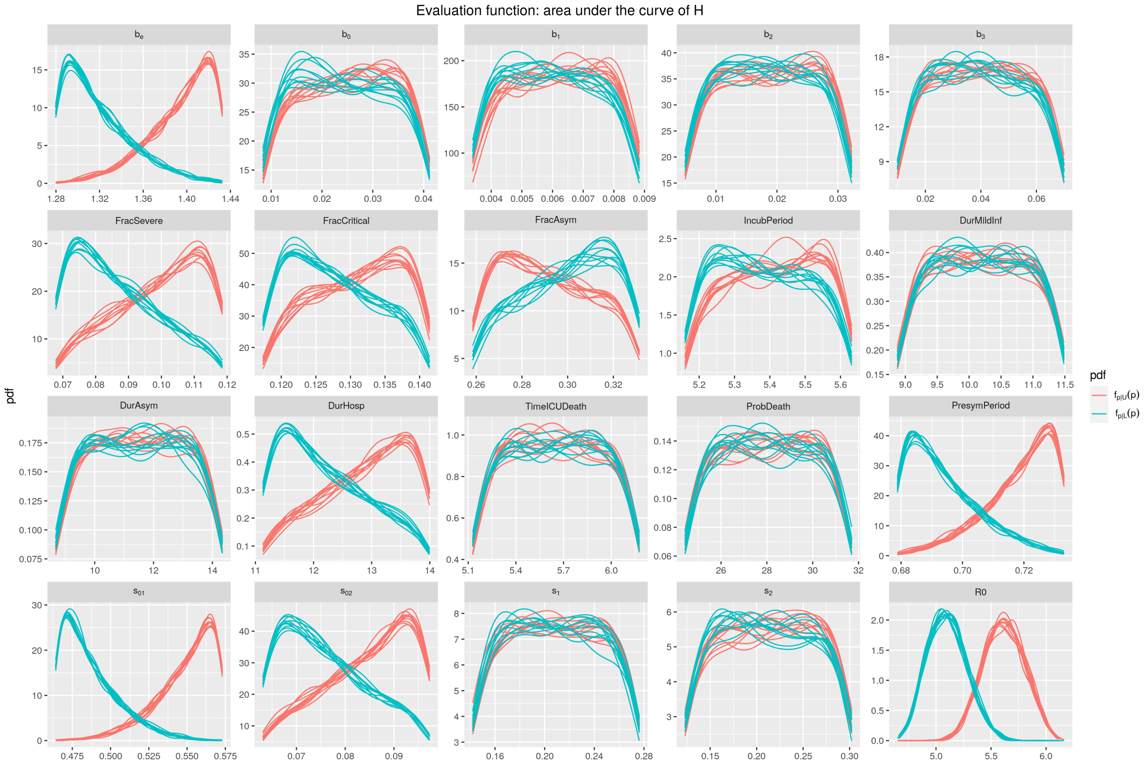

Figure S1: Umbria case. Conditional pdfs of model parameters after running Conditional Robustness Analysis (CRA) using as evaluation function the area under the curve of H. The time simulation set in the CRA algorithm is equal to 110 days (d). Parameters with a great separation of the two pdfs are those with an higher MIRI value.

strong impact on the area under the curve of H variable.

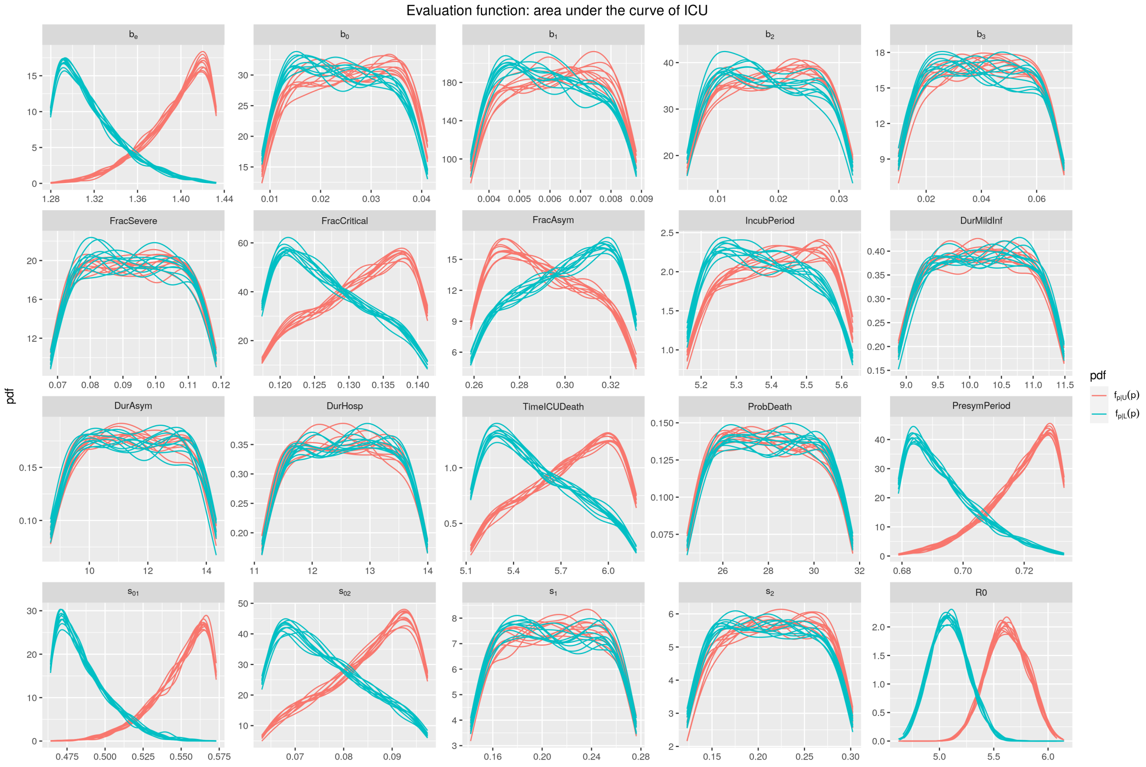

Figure S2: Umbria case. Conditional pdfs of model parameters after running the CRA using as evaluation function the area under the curve of ICU. The time simulation set in the CRA algorithm is equal to 110d.

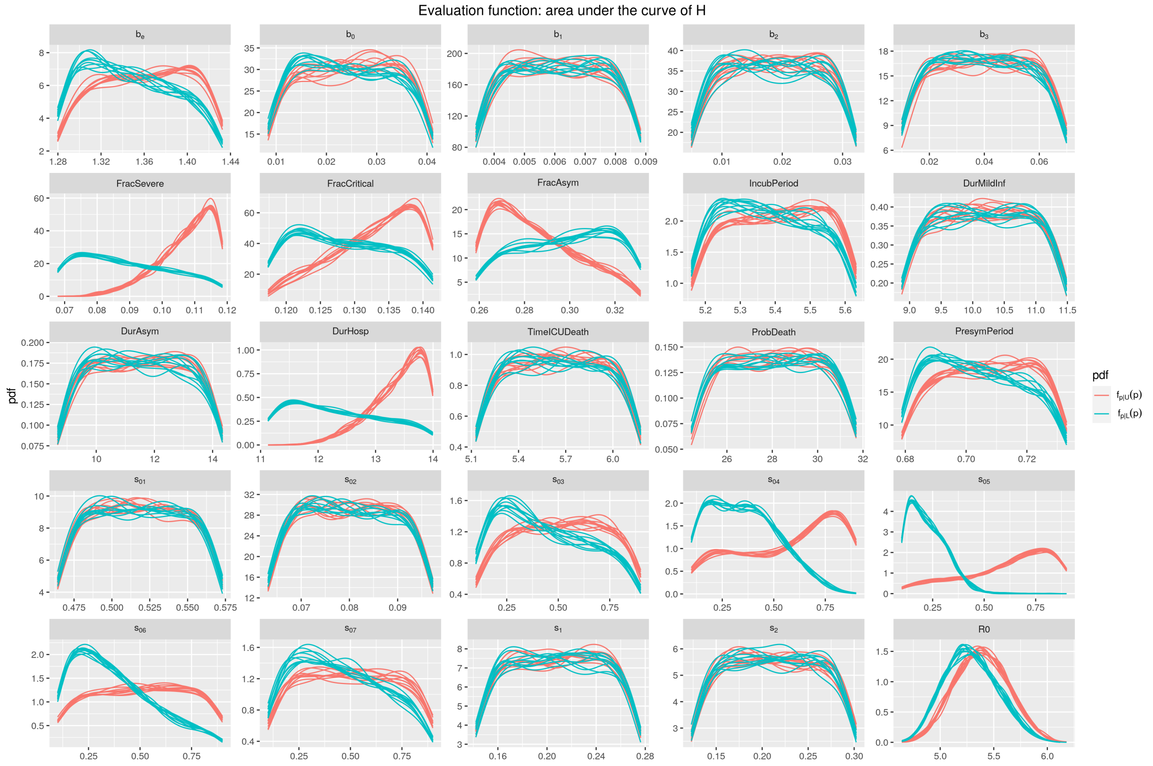

Figure S3: Umbria case. Conditional pdfs of model parameters after running the CRA using as evaluation function the area under the curve of H. The time simulation set in the CRA algorithm is equal to 250d. Five more parameters (s_{03}, s_{04}, s_{05}, s_{06} and s_{07}) are included in order to take into account the different lock-down measures adopted by the Italian government after the beginning of May.

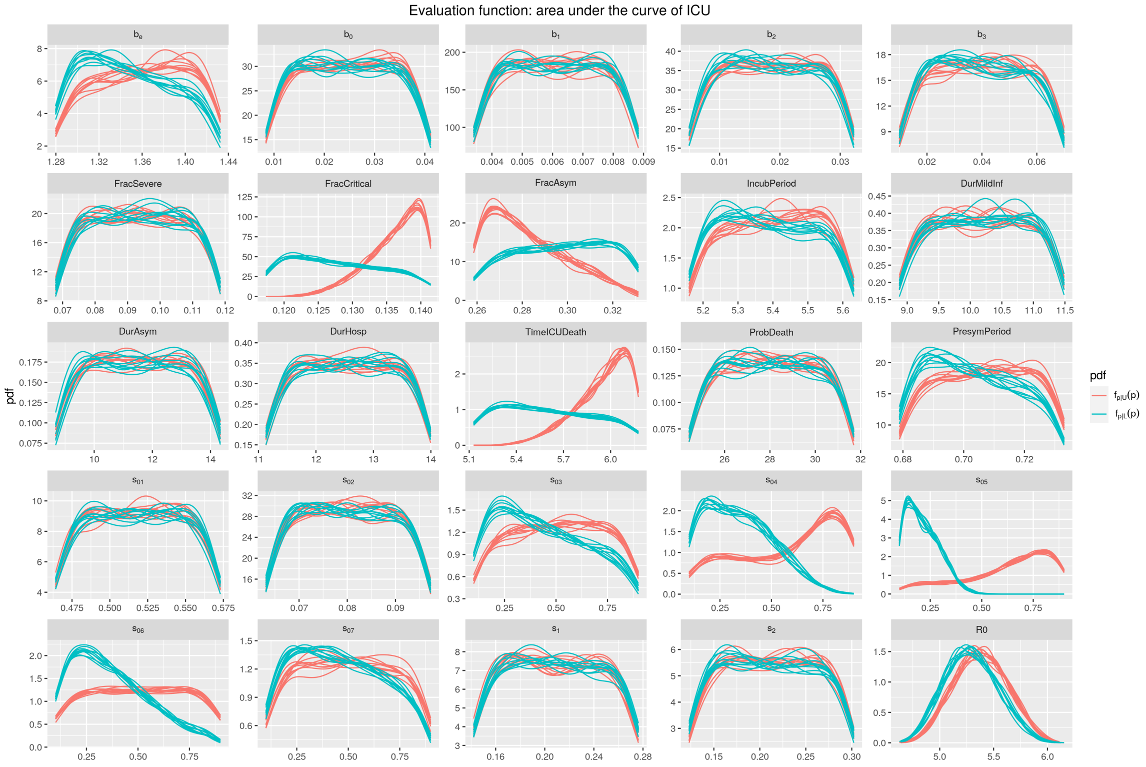

Figure S4: Umbria case. Conditional pdfs of model parameters after running the CRA using as evaluation function the area under the curve of ICU. The time simulation set in the CRA algorithm is equal to 250d.

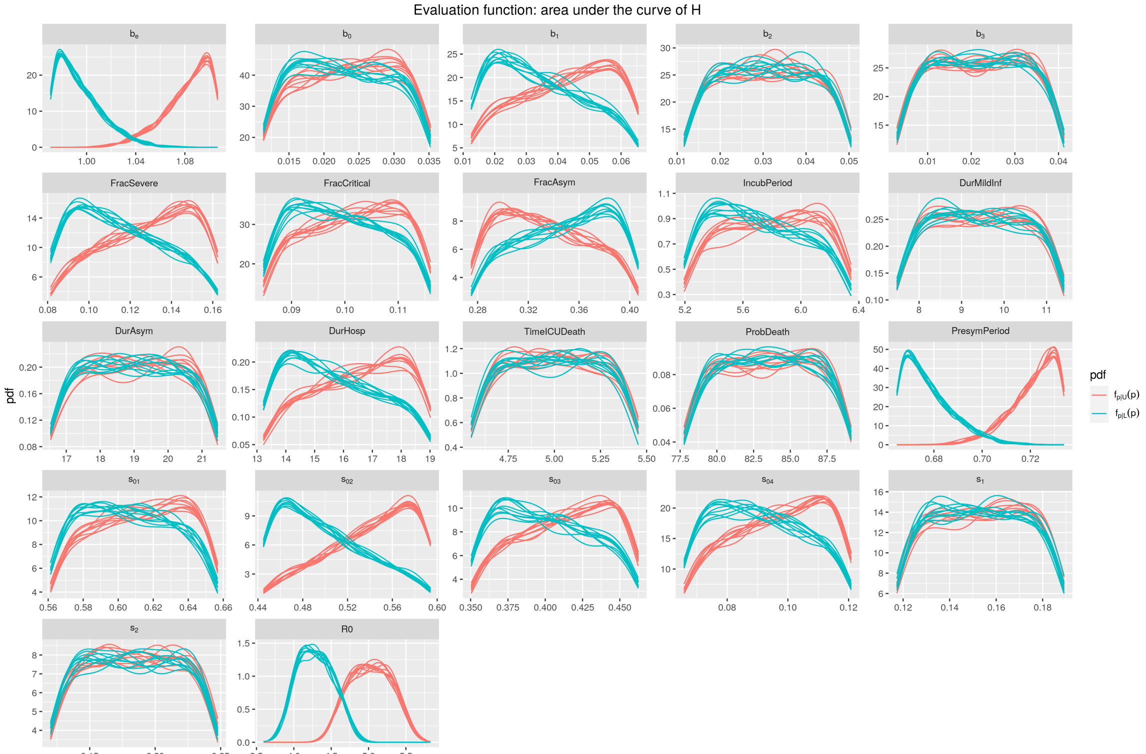

Figure S5: Italy case. Conditional pdfs of model parameters after running Conditional Robustness Analysis (CRA) using as evaluation function the area under the curve of H. The time simulation set in the CRA algorithm is equal to 110 days (d). Parameters with a great separation of the two pdfs are those with an higher MIRI value.

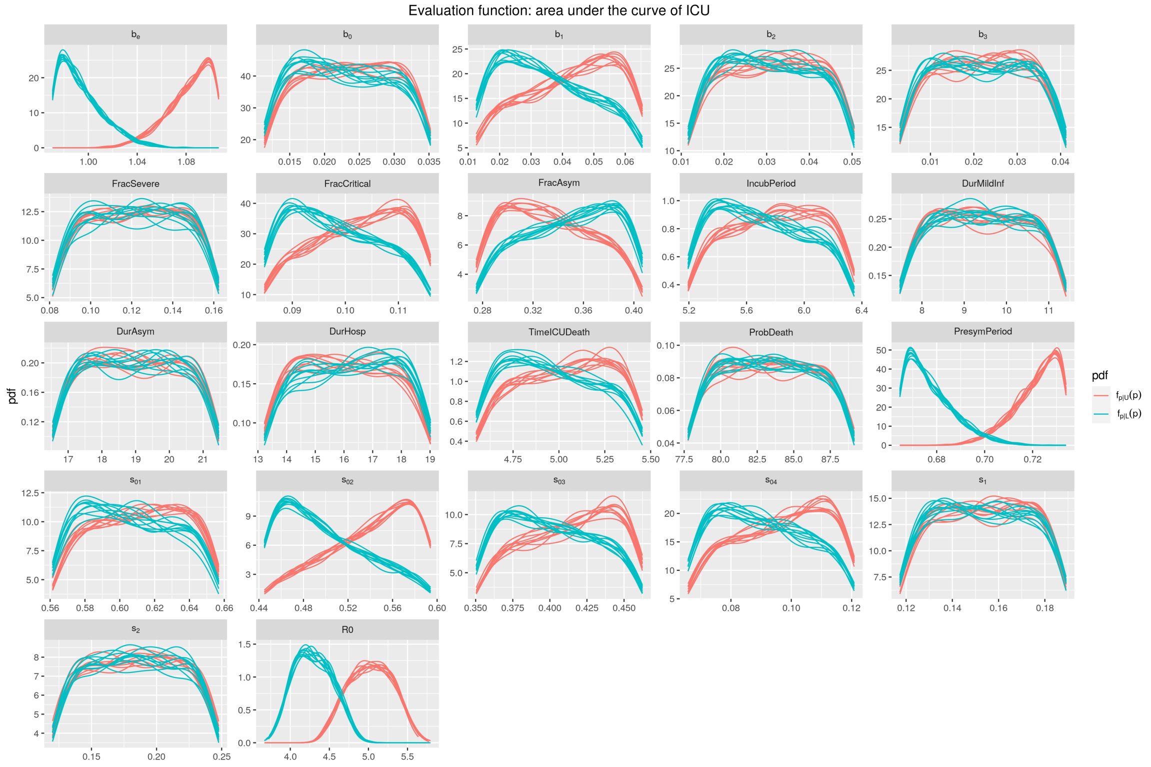

Figure S6: Italy case. Conditional pdfs of model parameters after running the CRA using as evaluation function the area under the curve of ICU. The time simulation set in the CRA algorithm is equal to 110d.

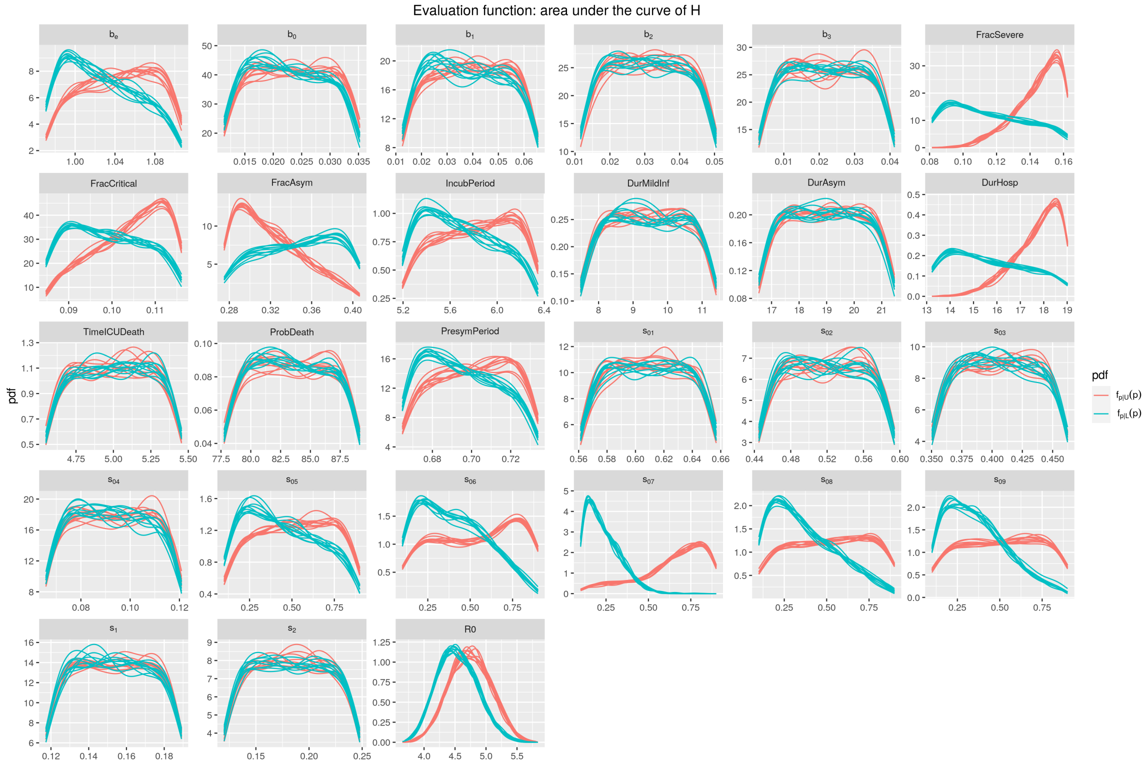

Figure S7: Italy case. Conditional pdfs of model parameters after running the CRA using as evaluation function the area under the curve of H. The time simulation set in the CRA algorithm is equal to 300d. Five more parameters (s_{05}, s_{06}, s_{07}, s_{08} and s_{09}) are included in order to take into account the different lock-down measures adopted by the Italian government after the beginning of May.

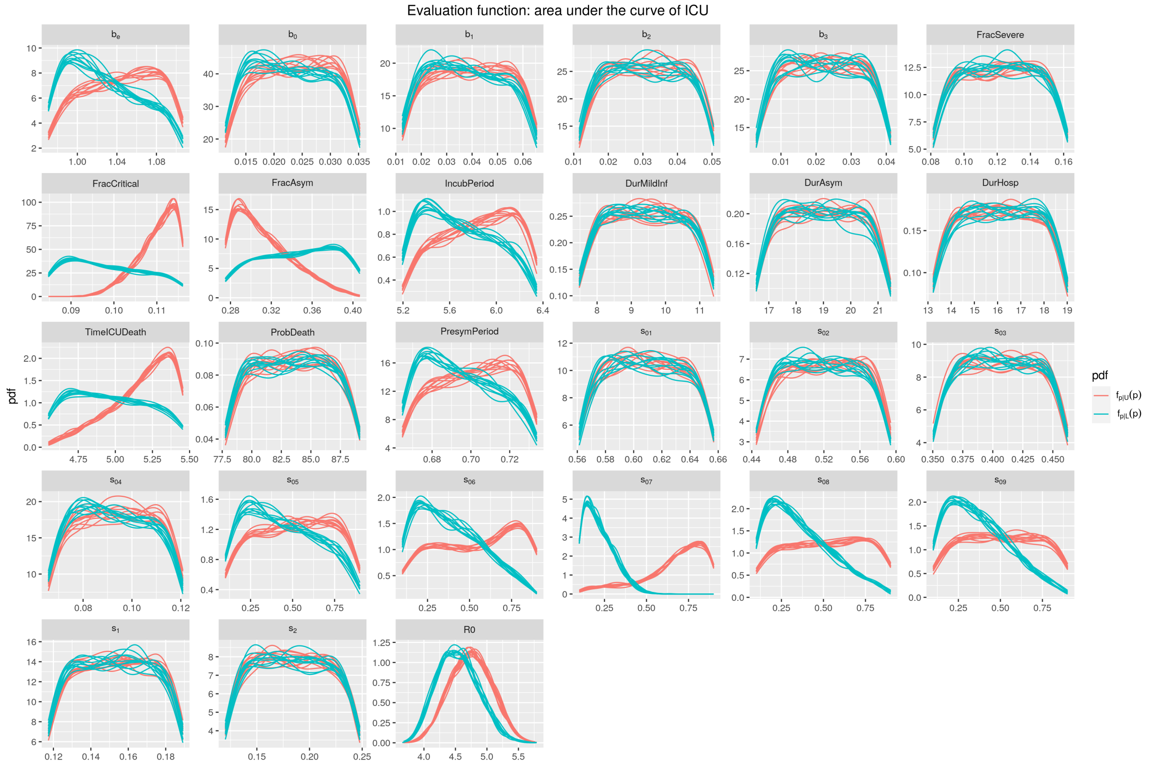

Figure S8: Italy case. Conditional pdfs of model parameters after running the CRA using as evaluation function the area under the curve of ICU. The time simulation set in the CRA algorithm is equal to 300d.
