# Supplementary figures and images for: Mathematical Modeling and Robustness Analysis to Unravel COVID-19 Transmission Dynamics: The Italy Case
Source: Biology (Basel). 2020 Nov 11;9(11):394. doi: 10.3390/biology9110394 (PMC7697740; doi:10.3390/biology9110394)

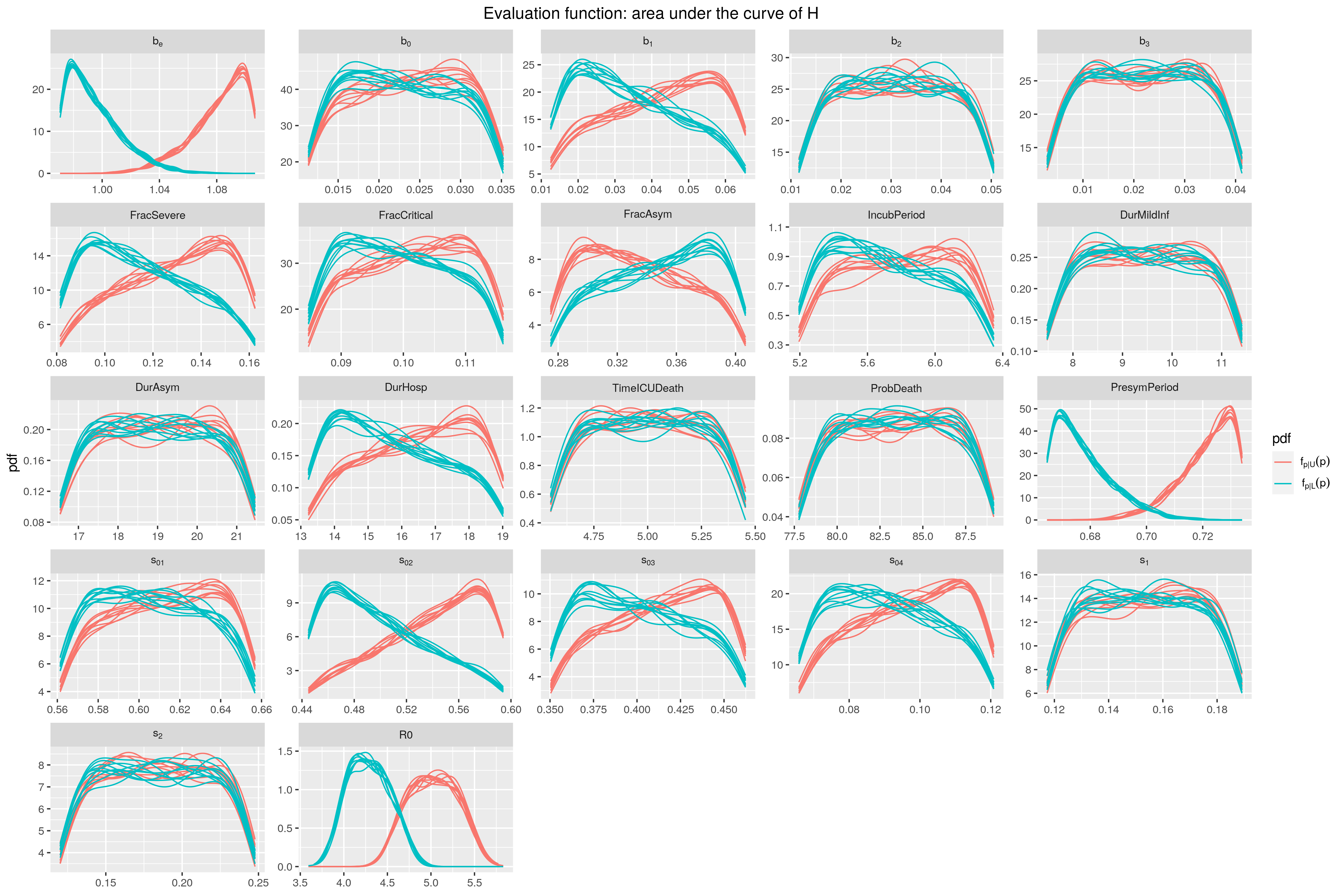

Supplement: Supplementary file 1 [file biology-09-00394-s001.zip › supplementary_file_review/img/pdf_param_area_I2_ITALY_4Nr_90perc_time110.png]

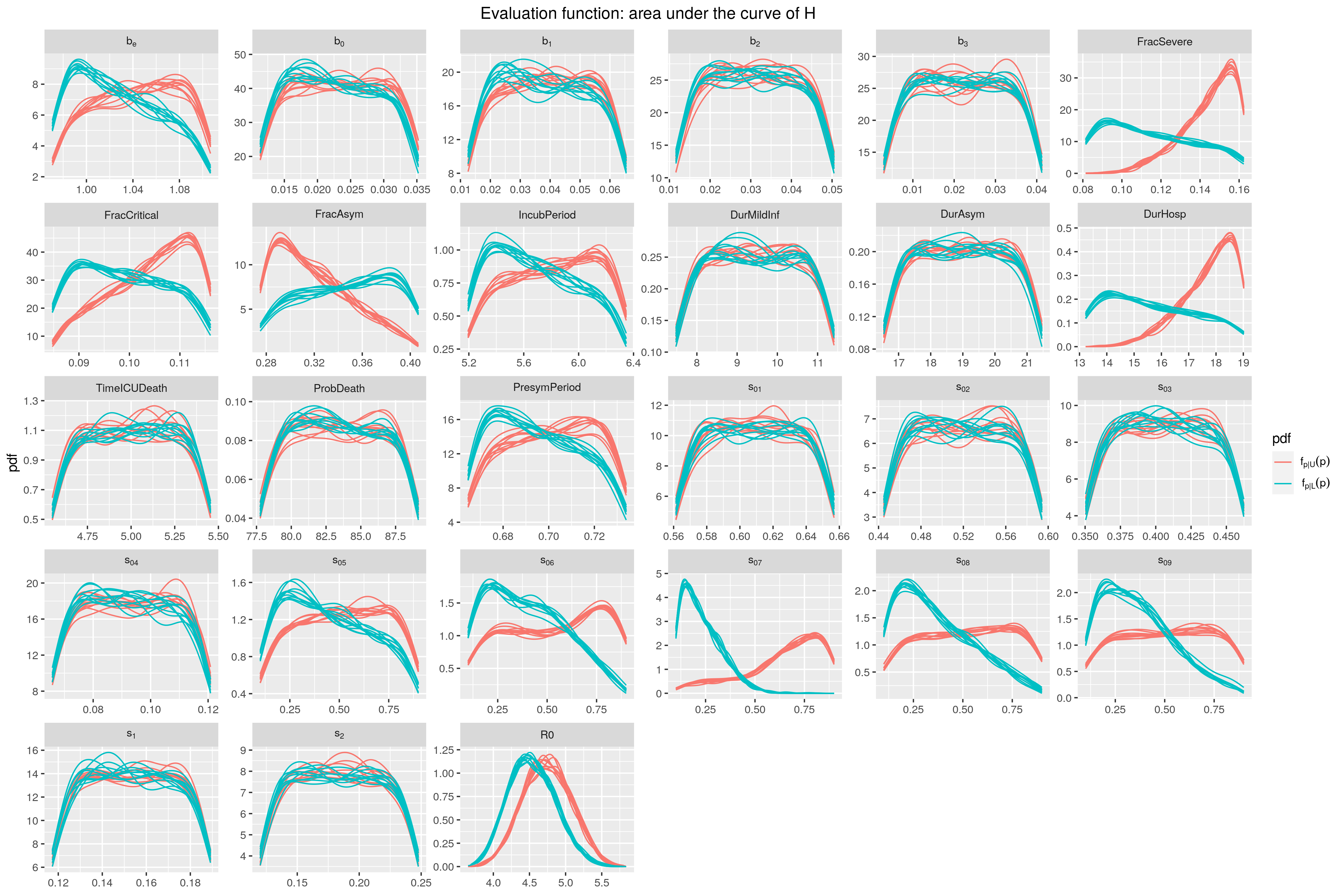

Supplement: Supplementary file 1 [file biology-09-00394-s001.zip › supplementary_file_review/img/pdf_param_area_I2_ITALY_time300.png]

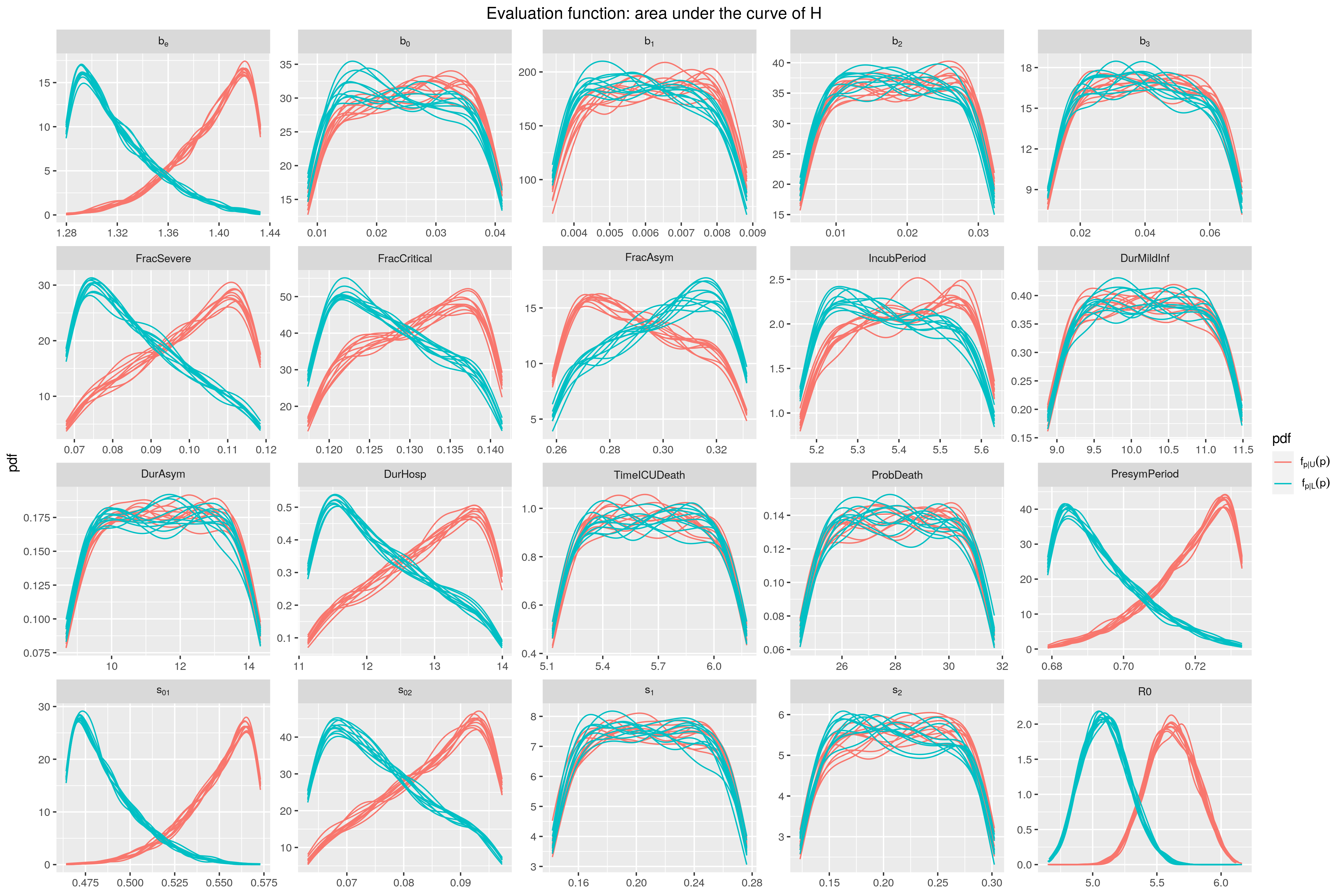

Supplement: Supplementary file 1 [file biology-09-00394-s001.zip › supplementary_file_review/img/pdf_param_area_I2_UMBRIA_time110.png]

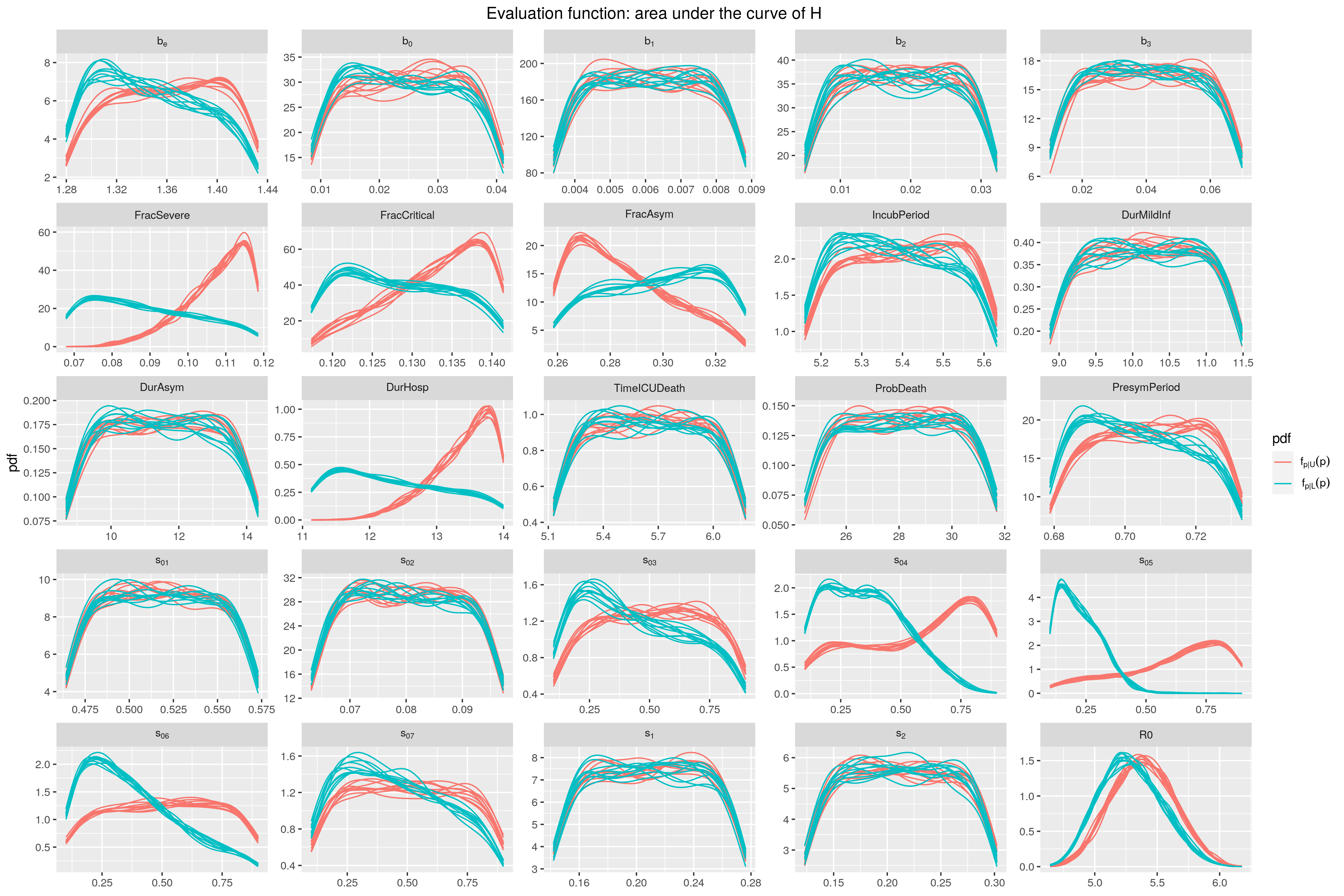

Supplement: Supplementary file 1 [file biology-09-00394-s001.zip › supplementary_file_review/img/pdf_param_area_I2_UMBRIA_time250.png]

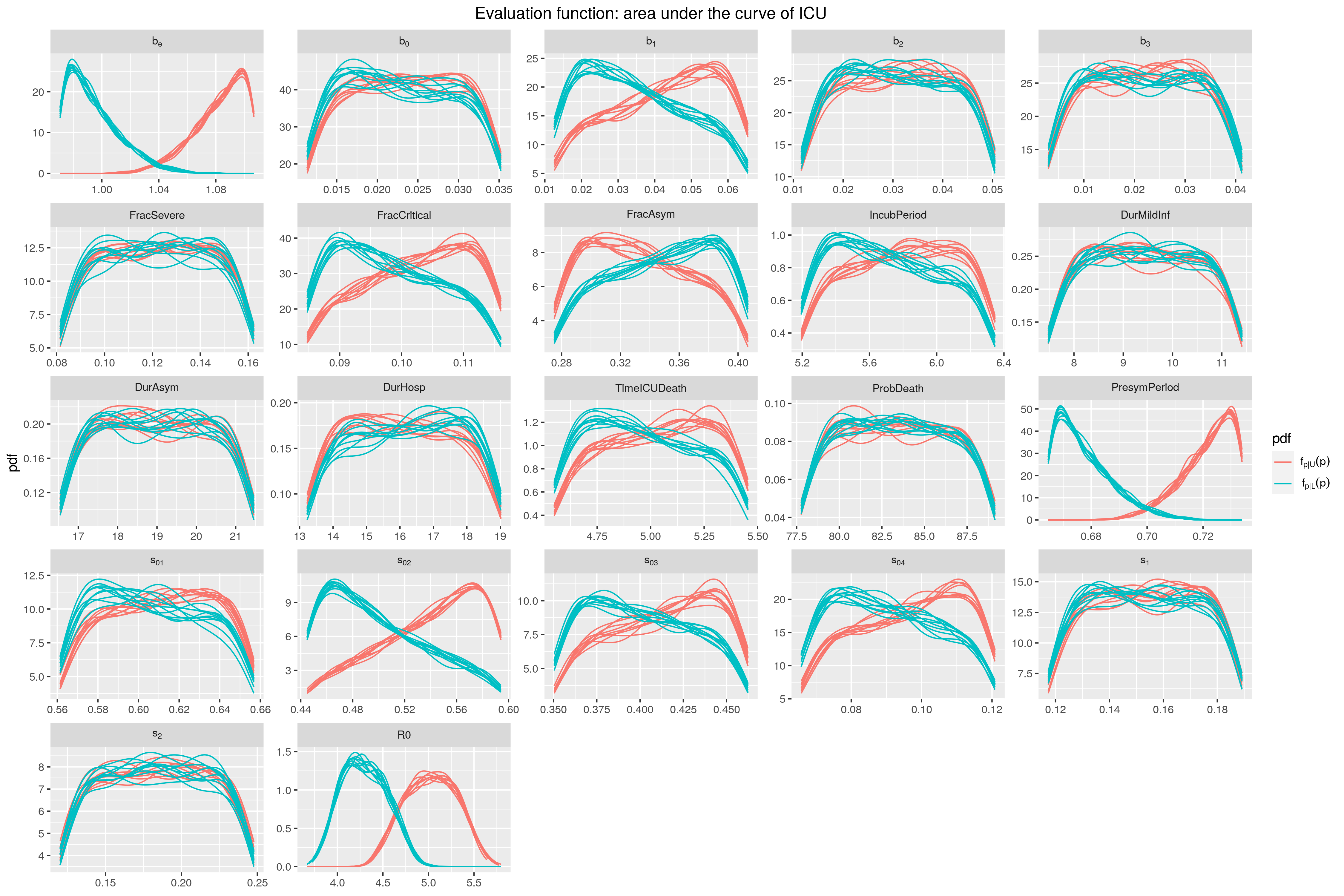

Supplement: Supplementary file 1 [file biology-09-00394-s001.zip › supplementary_file_review/img/pdf_param_area_I3_ITALY_4Nr_90perc_time110.png]

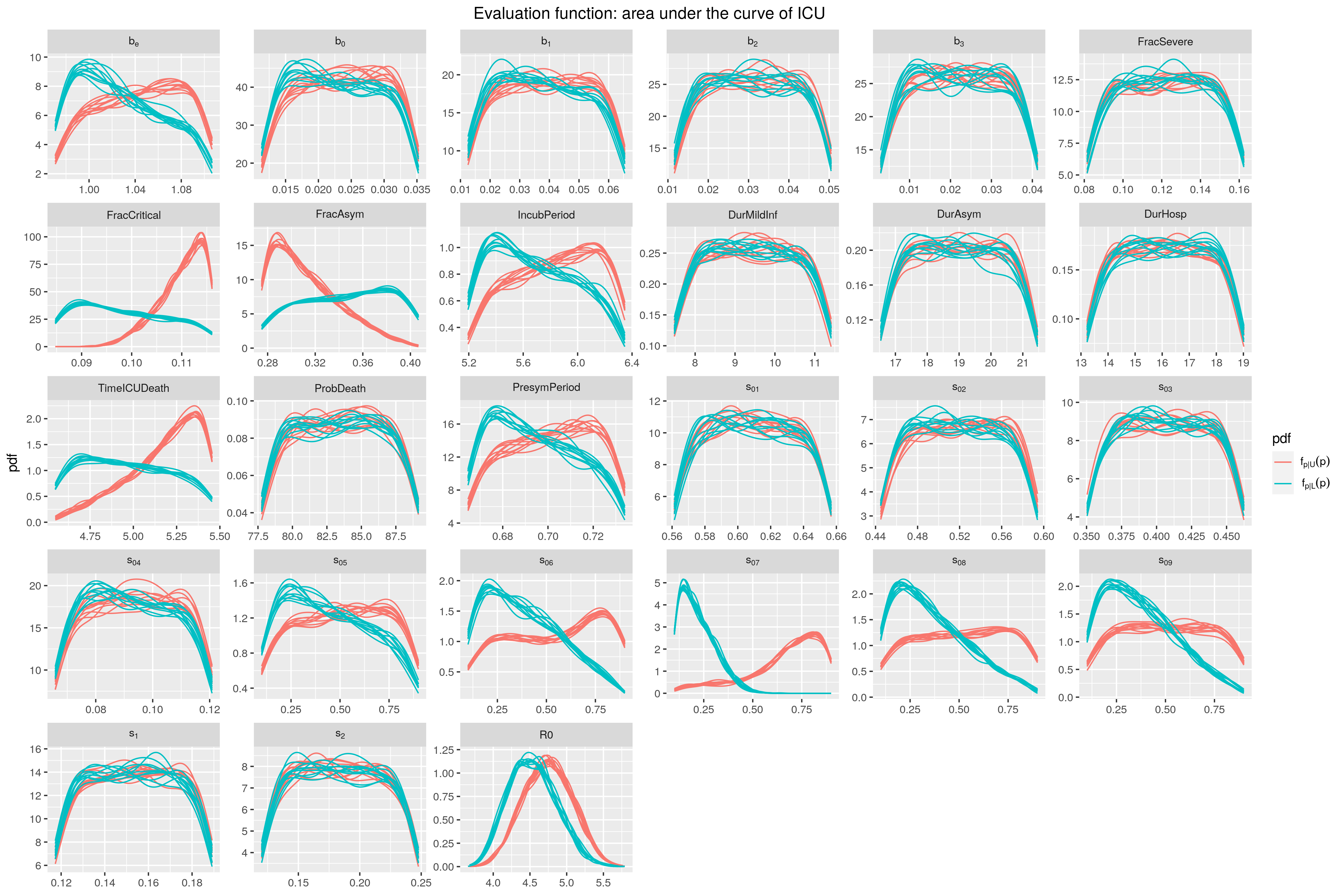

Supplement: Supplementary file 1 [file biology-09-00394-s001.zip › supplementary_file_review/img/pdf_param_area_I3_ITALY_time300.png]

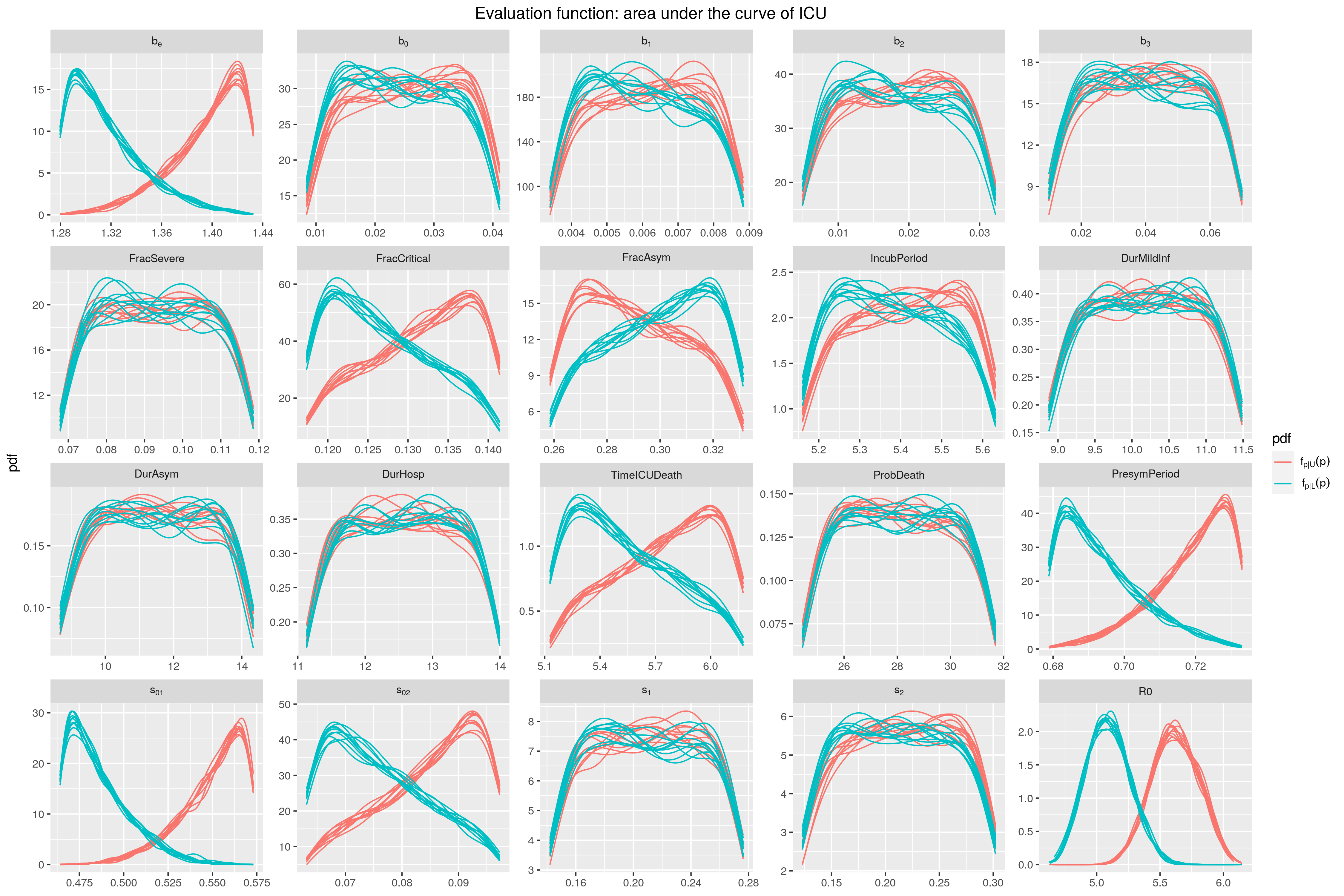

Supplement: Supplementary file 1 [file biology-09-00394-s001.zip › supplementary_file_review/img/pdf_param_area_I3_UMBRIA_time110.png]

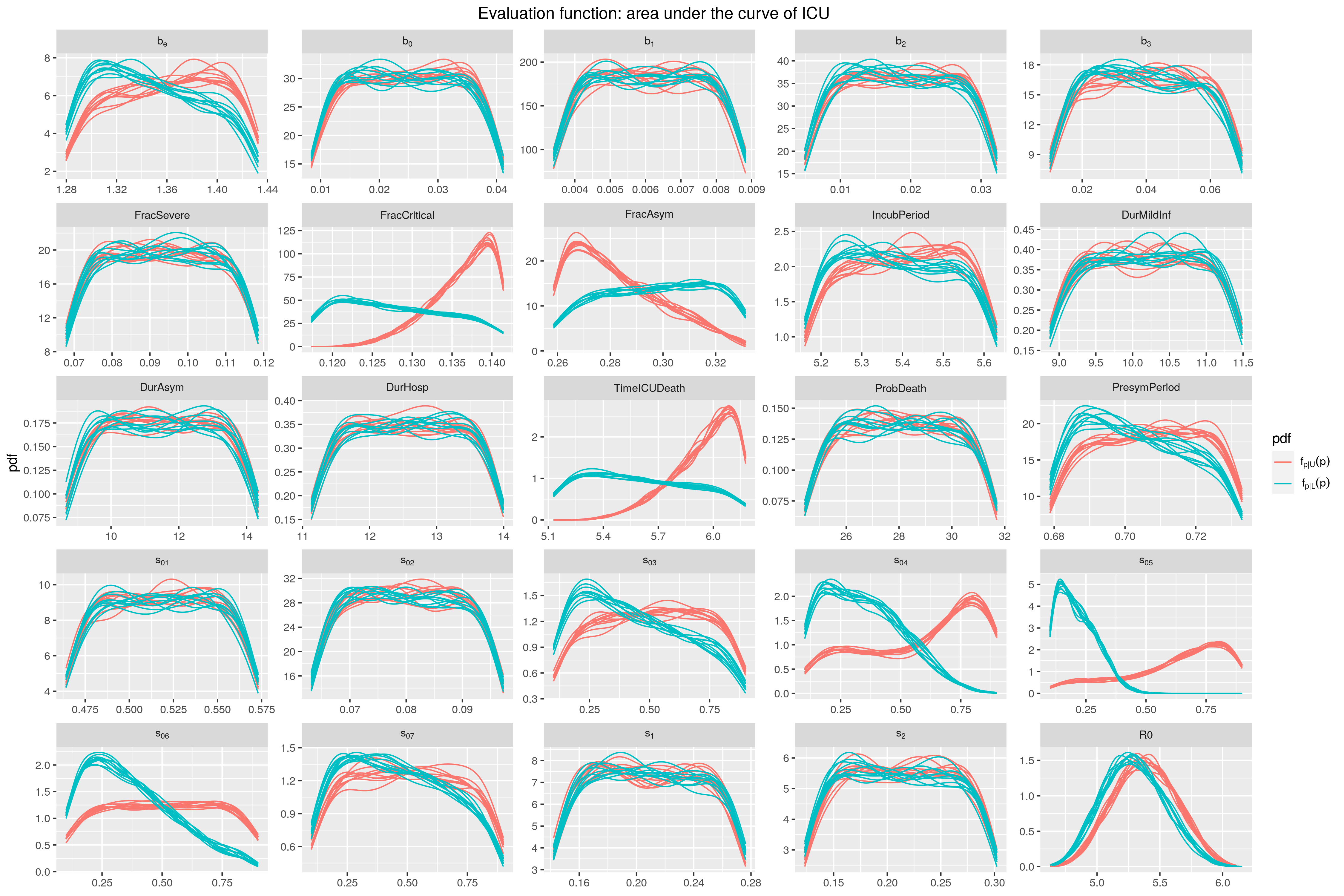

Supplement: Supplementary file 1 [file biology-09-00394-s001.zip › supplementary_file_review/img/pdf_param_area_I3_UMBRIA_time250.png]
